# Evidence for Coordinated Control of PM_2.5_ and O_3_: Long-Term Observational Study in a Typical City of Central Plains Urban Agglomeration

**DOI:** 10.3390/toxics13050330

**Published:** 2025-04-23

**Authors:** Chenhui Jia, Guangxuan Yan, Xinyi Yu, Xue Li, Jing Xue, Yanan Wang, Zhiguo Cao

**Affiliations:** 1 Key Laboratory for Yellow River and Huai River Water Environment and Pollution Control, Ministry of Education, School of Environment, Henan Normal University, Xinxiang 453007, China; jiachenhui@htu.edu.cn (C.J.); guangxuan.yan@htu.edu.cn (G.Y.); 15137362587@163.com (X.Y.); mixinyisnow@hotmail.com (X.L.); 2 Key Laboratory for Space Bioscience and Biotechnology, School of Life Sciences, Northwestern Polytechnical University, Xi’an 710072, China; xuejing0089@nwpu.edu.cn; 3 Department of Civil and Environmental Engineering, The Hong Kong Polytechnic University, Hong Kong, China

**Keywords:** ozone, PM_2.5_, long-term trend, random forest, observation-based model

## Abstract

Fine particulate matter (PM_2.5_) and Ozone (O_3_) pollution have emerged as the primary environmental challenges in China in recent years. Following the implementation of the Air Pollution Prevention and Control Action Plan, a substantial decline in PM_2.5_ concentrations was observed, while O_3_ concentrations exhibited an increasing trend across the country. Here, we investigated the long-term trend of O_3_ from 2015 to 2022 in Xinxiang City, a typical city within the Central Plains urban agglomeration. Our findings indicate that the hourly average O_3_ increased by 3.41 μg m^−3^ yr^−1^, with the trend characterized by two distinct phases (Phase I, 2015–2018; Phase II, 2019–2022). Interestingly, the increasing rate of O_3_ concentration in Phase I (7.89 μg m^−3^) was notably higher than that in Phase II (2.89 μg m^−3^). The Random Forest (RF) model was employed to identify the key factors influencing O_3_ concentrations during the two phases. The significant dropping of PM_2.5_ in Phase I could be responsible for the O_3_ increase. In Phase II, the reductions in nitrogen dioxide (NO_2_) and unfavorable meteorological conditions were the major drivers of the continued increase in O_3_. The Observation-Based Model (OBM) was developed to further explore the role of PM_2.5_ in O_3_ formation. Our results suggest that PM_2.5_ can influence O_3_ concentrations and the chemical sensitivity regime through heterogeneous reactions and changes in photolysis rates. In addition, the relatively high concentration of PM_2.5_ in Xinxiang City in recent years underscores its significant role in O_3_ formation. Future efforts should focus on the joint control of PM_2.5_ and O_3_ to improve air quality in the Central Plains urban agglomeration.

## 1. Introduction

Tropospheric Ozone (O_3_) is a typical secondary gaseous pollutant and the third most significant greenhouse gas (IPCC, 2021). It has a profound impact on human health, ecosystem stability, and vegetation productivity [1]. In recent years, O_3_ pollution has emerged as a major environmental issue in the urban areas of China. Observational data indicate that ground-level O_3_ concentrations have been rising nationwide [2]. For instance, Wang et al. reported that the maximum daily 8 h average (MDA8) O_3_ level increased by 2.6 μg m^−3^ yr^−1^ in the warm season (April–September) from 2013 to 2020 [3]. This upward trend in O_3_ concentrations was similarly observed in many megacities in China, such as Beijing [4], Shanghai [5], Sichuan Basin [6], and other cities [7,8,9]. However, the long-term trend of O_3_ concentrations in the Central Plains urban agglomeration remains relatively deficient at present.

In the troposphere, O_3_ is formed through complex radical chain reactions involving the oxidation of volatile organic compounds (VOCs) in the presence of nitrogen oxides (NO_x_ = NO_2_ + NO) under sunlight [10]. The rising trend in O_3_ concentration is influenced by a variety of factors, including increased global O_3_ background concentrations, the changes in meteorological conditions, and shifts in chemical regime due to various regulations affecting NO_x_ and VOCs emissions [11,12]. Although meteorological conditions and emission changes have been dominant drivers in recent O_3_ increases, their contributions have varied across different periods. Liu et al. [2] revealed that the impact of anthropogenic emissions on the O_3_ rise from 2017 to 2020 (1.2 μg m^−3^) was much lower than that during 2013–2017 (5.2 μg m^−3^) in China. In addition, the O_3_ concentration can be highly sensitive to the meteorological conditions in the given phases and periods. For instance, the meteorological conditions in May 2020 led to a significant increase of O_3_ by 26.8 μg m^−3^ compared to May 2019 in the Sichuan Basin [6]. Factors such as temperature, relative humidity, radiation intensity, wind speed, and wind direction were regarded as the main factors affecting O_3_ formation [13,14,15,16,17]. However, the key meteorological factors vary across different regions. According to Weng et al. [18], surface solar radiation is a primary determinant of O_3_ fluctuations in the Yangtze River Delta (YRD) and Sichuan Basin, while temperature is identified as the most important meteorological variable in the Beijing–Tianjin–Hebei (BTH) region.

Aerosols exert a complex influence on the O_3_ production rate through heterogeneous reactions, alterations in photolysis rates, and modifications to the boundary layer [19]. The “aerosol inhibited” regime in O_3_ formation, where heterogeneous reactions on aerosol particles predominantly lead to HO_2_ loss, has been identified through chemical transport modeling [20]. The enhancement of HO_2_ due to the dropping of aerosols has been recognized as a key driver for the increasing summertime O_3_ concentration in the North China Plain from 2013 to 2017 [21,22]. Furthermore, a study by Shao et al. [23] revealed that O_3_ formation in Beijing increased by 37% from 2006 to 2016 following a reduction in PM_2.5_ levels. Consequently, the reduction in PM_2.5_ concentrations could offset the effectiveness of traditional O_3_ precursor (VOC and NO_x_) control strategies under the “aerosol inhibited” photochemical O_3_ regime [3]. Hence, understanding O_3_ formation mechanisms and identifying the key factors are crucial for accurately managing O_3_ pollution, not only in China but also globally.

Machine learning techniques, such as artificial neural networks, random forest (RF), and the convolutional neural network, have been widely used in atmospheric research [24,25,26,27,28]. Among these methods, RF is employed to account for the nonlinear interactions between different input parameters without assuming any specific relationships [29]. Numerous studies [4,18,27,29,30] have demonstrated the efficacy of the RF model in predicting O_3_ levels and identifying primary factors influencing O_3_ formation. However, the interpretability of results from the RF model is limited due to its “black box” nature. As a complementary method, the observation-based model (OBM) coupled with the Master Chemical Mechanism (MCM) serves as an effective tool for investigating atmospheric photochemistry mechanisms. The MCM has been widely used to investigate in situ O_3_ formation processes and the sources of radicals [31,32,33,34,35,36]. However, OBM-MCM relies heavily on detailed observation data and is limited in its ability to conduct long-term and large-scale O_3_ pollution research.

Xinxiang City, located in the northern region of Henan Province, is a rapidly developing city within the Central Plains urban agglomeration. As a member of the “2 + 26” city cluster, which serves as a major air pollution transmission channel in the Beijing–Tianjin–Hebei region, Xinxiang suffered the severe haze pollution. In recent years, the exacerbation of O_3_ pollution has emerged as a critical environmental challenge. However, the quantitative relationship between reductions in PM_2.5_ concentrations and concurrent increases in O_3_ remains unclear. To investigate the relationship between PM_2.5_ and O_3_, this study proposes a multi-temporal analytical framework integrating RF and OBM.

By integrating long-term continuous monitoring data (2015–2022) with short-term intensive high-density observations, this study aims to quantify long-term key drivers and elucidate the underlying mechanisms in O_3_ pollution in Xinxiang City. Firstly, the long-term trend and seasonal variation of O_3_ during this period were explored by using hourly observations of O_3_ collected from the national monitoring network. Subsequently, the RF model was employed to investigate the factors influencing O_3_ levels and assign importance rankings to these factors. Finally, the OBM was utilized for illustrating the mechanism underlying the identified influencing factors in O_3_ formation. The results of this work are expected to provide insights beneficial for controlling O_3_ pollution in cities within the Central Plains urban agglomeration.

## 2. Materials and Methods

### 2.1. Data Sources

**Figure 1:**
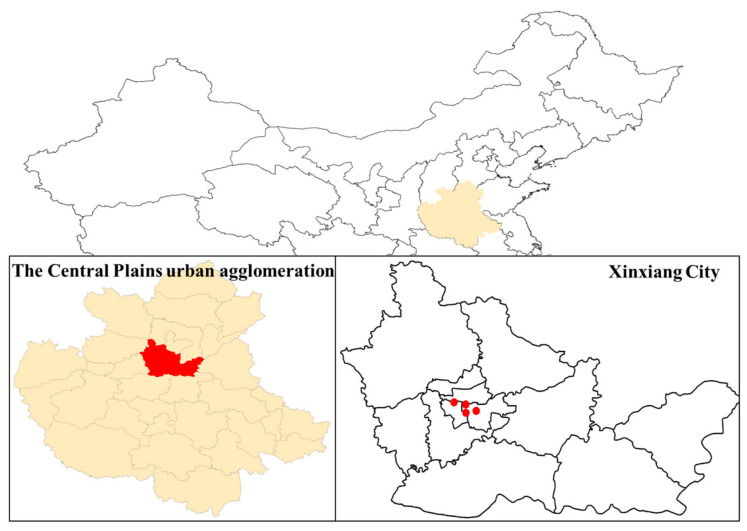
Location of the nation-controlled air quality automatic monitoring stations in Xinxiang City. The red dots represent four state-operated air quality automatic monitoring stations.

Xinxiang City has been equipped with four state-operated air quality automatic monitoring stations since 2015, which are strategically positioned primarily within the urban area (Figure 1). Hourly concentrations of air pollutants (including O_3_, NO_2_, CO, SO_2_, PM_2.5_, and PM_10_) from these four sites were obtained from the China National Environmental Monitoring Centre (http://www.cnemc.cn/, accessed on 16 May 2024), covering the period from 1 January 2015 to 31 December 2022. The pollutants data were normalized based on the change of atmospheric conditions before (273.15 K, 1 atm) and after (298.15 K, 1 atm) September 2018.

The meteorological data from 1 January 2015 to 31 December 2022 were obtained from the ERA5 datasets of the European Centre for Medium-Range Weather Forecasts (ECMWF) (https://cds.climate.copernicus.eu/cdsapp#!/dataset/reanalysis-era5-single-levels?tab=overview, accessed on 19 May 2024). The research area (34°55′–35°50′ E; 113°30′–115°01′ N) covered the entire Xinxiang City. The hourly resolution of significant meteorological variables involving the O_3_ formation mechanism with a spatial resolution of 0.25° × 0.25° was utilized in our study, including a 10 m u-component of wind, 10 m v-component of wind, 2 m dewpoint temperature, 2 m temperature, boundary layer height, surface net solar radiation, surface pressure, total cloud cover, and total precipitation. The detailed information of these variables can be found in Table 1.

The field measurement campaign was also conducted from 1 June to 31 June in 2021. The sampling site was located at the Xinxiang Municipal Party School (35.29° N, 113.93° E), a typical urban area. The gaseous pollutants, including O_3_, NO_2_, NO, SO_2_, CO, and NMVOCs, were measured in our study. The Model 42*i*, Model 48*i*, Model 43*i*, and Model 49*i* (Thermo Fisher Scientific, Waltham, MA, USA) were used for online measurements of NO_x_ (NO_2_, NO), SO_2_, CO, and O_3_. The hourly NMVOCs concentrations, including alkanes, alkenes, alkynes, aromatics, and oxygenated compounds were measured by GC-FID/MS (TH-300B, Wuhan Tianhong Environmental Protection Industry Co., Ltd., Wuhan, China).

### 2.2. Random Forest Model

The RF model is an ensemble learning algorithm with high accuracy and a strong ability to avoid overfitting. Here, the RF model was developed to predict the concentrations of O_3_ and identify critical variables in O_3_ formation. The performance of RF depends on hyperparameters. Details of all parameters tuned for the RF model are presented in Table S1. The randomForest package for the R software (version 4.2.3) is used for analyses and validation processes in our study.

For the RF model, the in situ observation pollutants concentrations and meteorology factors were selected as input variables. Due to a lack of long-term hourly observation, VOCs were excluded from the input parameters in this study. According to previous studies [37,38,39,40], the variability of surface O_3_ was well-explained by the ML algorithm with meteorological information alone, particularly in the VOC-limited regime. Like many other urban areas in China, O_3_ production in Xinxiang City is generally in the VOC-limited regime. Therefore, it is reasonable to simulate O_3_ using a supervised RF model without considering the VOC concentration.

The datasets were randomly divided into training and testing subsets at a ratio of 7:3. The fivefold cross-validation method was used to evaluate the performance of the RF model [27] (Figure S1). The relative importance of the input variables was ranked by calculated variable importance scores, represented as the aggregated increase in the mean squared errors (%IncMSE). The mean squared errors were calculated by the RF model by randomly assigning values to each input variable. The variables with a higher importance score (%IncMSE) had a more significant impact on O_3_ formation.

### 2.3. Observation-Based Model

OBM incorporated with MCM v3.3.1 was built to investigate the chemical mechanism of how PM_2.5_ affects the formation of O_3_. The detailed description of the gas-phase chemical processes by the MCM displays that it was involved in methane and 142 non-methane VOCs [41]. To establish a direct relationship between PM_2.5_ concentrations and O_3_ formation, the OBM considered the heterogeneous reactions and variations in photolysis rates. The aerosol optical depth (AOD) could be calculated by the PM_2.5_ concentration [23,42] using Equation (1):

(1) $$\frac{AOD}{H}={PM}_{2.5}\times K\times f\left(RH\right)\times 10^{-6}$$

where H represents the atmosphere boundary layer height; f(RH) denotes the hygroscopic growth factor, which is determined by relative humidity (RH), and K is the given parameter.

The calculated AOD was used to quantify the hourly photolysis rates of NO_2_ (*J*NO_2_) [43,44], thus establishing a direct link between PM_2.5_ concentration and photolysis rates (see details in the Supplemental Information). The photolysis rates (*J_i_*) of other species were calculated by the solar zenith angle (*SZA*) and built-in parameters (*L_i_*, *M_i_*, and *N_i_*) [45]; see Equation (2):

(2) $$J_{i}=L_{i}\times cos\left(SZA\right)\times M_{i}\times exp\left(–N_{i}\times sec\left(SZA\right)\right)$$

The photolysis rates would be further scaled according to the calculated photolysis rates of NO_2_ (*J*NO_2_) based on the PM_2.5_ concentration.

The heterogeneous reaction of HO_2_ was assumed to be the first order reaction [21,46], and the reaction constant (k) could be calculated by Equation (3):

(3) $$k={-\left(\frac{r}{D_{g}}+\frac{4}{{\gamma HO}_{2}}\times {vHO}_{2}\right)}^{-1}\times S_{aero}$$

where r, Dg, and *v*HO_2_ were the surface-weighted particle radius, gas phase diffusion coefficient, and mean molecular speed of HO_2_, respectively. The relevant values of these parameters were selected according our previous study [33]. γHO_2_ was the uptake coefficient of HO_2_ on aerosols, ranging from 0.02 to 0.2. The O_3_ concentration under different γHO_2_ was tested by OBM (Figure S1). In our study, the maximum γHO_2_ value of 0.2 was adopted to magnify the effect by the model according to Shao et al.’s study [23]. S_aero_ was the aerosol surface concentration, which is calculated by the PM_2.5_ concentration (further details are provided in the Supplemental Information).

The observed and calculated data, including pollutant concentrations (CO, SO_2_, NO_x_ (NO, NO_2_), and NMVOCs) and meteorological factors (relative humidity, temperature, pressure, and the photolysis rates in related species) were subjected to the model constraints. The time resolution of the input parameters was averaged or interpolated to 1 h.

### 2.4. Model Evaluation

The mean bias (MB), root mean squared error (RMSE), and index of agreement (IOA) were used to assess the model (RF and OBM) performance based on the observed (*O_i_*) and simulated (*S_i_*) hourly O_3_ values according to the following equations:

(4) $$MB=\frac{\sum_{i=1}^{N}\left(S_{i}-O_{i}\right)}{N}\;$$

(5) $$RMSE=\sqrt{\frac{\sum_{i=1}^{N}{\left(S_{i}-O_{i}\right)}^{2}}{N}}$$

(6) $$IOA=1-\frac{\sum_{i=1}^{N}{\left(O_{i}-S_{i}\right)}^{2}}{\sum_{i=1}^{N}{\left(\left|O_{i}-\bar{O}\right|+\left|S_{i}-\bar{O}\right|\right)}^{2}}$$

where $\bar{O}\;$ is the mean concentration of the observed O_3_.

## 3. Results and Discussion

### 3.1. O_3_ Pollution Profiles

#### 3.1.1. Long-Term Trend of O_3_ and Related Pollutants

**Figure 2:**
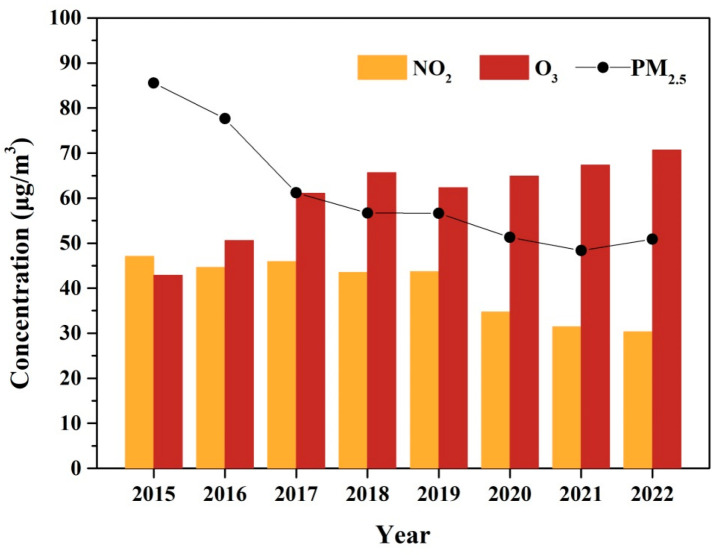
The annual trend of O3, NO2, and PM2.5 during 2015–2022.

The year variations of 1 h O_3_ concentrations and related pollutants (NO_2_ and PM_2.5_) in Xinxiang City are presented in Figure 2. The O_3_ concentration exhibited an increasing trend from 2015 to 2022, with an average growth rate of 3.41 μg m^−3^ yr^−1^. The similar upward trends in O_3_ concentrations over the past 1–2 decades have been observed in other Chinese urban areas, such as Beijing [47], Shanghai [48], the Sichuan Basin [6], the Pearl River Delta [49], and various other Chinese urban sites [2,7]. In contrast, the concentrations of PM_2.5_ and NO_2_ showed significant declines from 2015 to 2022. This reduction is attributed to the stringent implementation of clean air policies in China, including the Air Pollution Prevention and Control Action Plan (2013–2017) and the Three-Year Action Plan for Winning the Blue Sky Defense Battle (2018–2020) [3]. The former plan focused primarily on reducing particulate matter, while the latter emphasized the coordinated control of NO_x_ and VOCs, with a targeted 10% reduction in VOC emissions [50].

The increasing O_3_ trend could be further separated into two phases (Phase I, 2015–2018; Phase II, 2019–2022) based on the different increasing rate. During Phase I, the average 1-hourly O_3_ concentration increased at a rate of 7.89 μg m^−3^ yr^−1^. The average annual concentration of PM_2.5_ was at a high level, and had a significant decrease (from 85.54 to 56.70 μg m^−3^). However, no significant changes were observed in NO_2_ concentration during Phase I. In contrast, during Phase II, the increase rate of O_3_ was 2.76 μg m^−3^ yr^−1^, which was much smaller than that in Phase I. The concentration of PM_2.5_ was also at the high level (approximately 50 μg m^−3^), although it experienced a relatively smaller decrease compared to Phase I. By contrast, the concentration of NO_2_ had an obvious decreasing tendency in Phase II.

NO_2_ was the important precursor in O_3_ formation through the “NO_x_ cycle”, exhibiting a non-linear relationship with O_3_ formation. Under the VOC-limited conditions, which were thought to prevail in urban China, decreasing NO_x_ would increase O_3_, while under NO_x_-limited conditions, reducing NO_x_ could decrease O_3_ concentrations [22]. The effect of PM_2.5_ on O_3_ formation was mainly by changing photolysis rates and heterogeneous chemical processes [23], with its influence heavily dependent on the level of the PM_2.5_ concentration. In Xinxiang City, O_3_ formation was under VOC-limited regimes alongside a high PM_2.5_ concentration. In the condition, reductions in both NO_x_ and PM_2.5_ can lead to increased O_3_ production. Hence, the decline in PM_2.5_ concentration could be a primary factor driving the rise in O_3_ in Phase I. The result was consistent with the results that a reduction of PM_2.5_ stimulated O_3_ production over the 2013–2017 periods in the North China Plain. The impact of PM_2.5_ controls on O_3_ formation likely weakened in Phase II due to the relatively minor reduction in PM_2.5_ concentrations. The unbalanced changing of the precursor concentration (VOC and NO_2_) might be the main reason for O_3_ increasing in Phase II.

#### 3.1.2. Seasonal Variation of O_3_ Pollution

**Figure 3:**
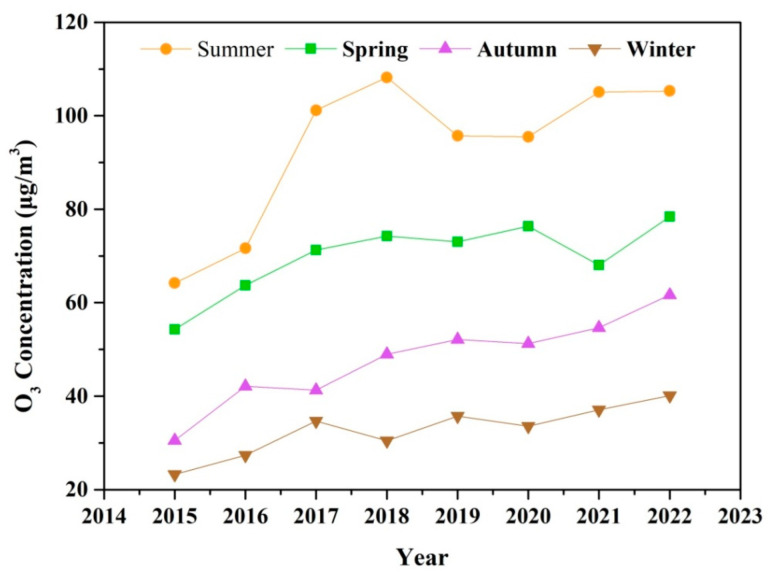
The seasonal variation of O_3_ pollution during 2015–2022.

The seasonal variation of O_3_ during 2015–2022 is shown in Figure 3. O_3_ concentrations exhibit pronounced seasonal patterns, peaking during the summer, and remaining at relatively lower levels in the winter. The rise in temperature and solar radiation intensity plays a critical role in photochemical formation of O_3_ in summer [51]. Enhanced photochemical production and the rapid cycling of RO_x_ radicals (OH + HO_2_ + RO + RO_2_) typically overcome the radical and NO titration in summer [52]. Consequently, the potential health hazards associated with O_3_ exposure are particularly significant during the warm season. According to the updated WHO Global Air Quality Guidelines (AQGs) from September 2021, the recommended peak season O_3_ concentration is lower than 60 μg m^−3^. However, the average concentration of O_3_ in summer and spring exceed the recommended threshold from 2015 to 2022. In autumn and winter, a steady increase in O_3_ concentration has been observed since 2018, with levels exceeding 60 μg m^−3^ in autumn 2022. The extension of the O_3_ pollution season from the warm season is a nationwide phenomenon in China [53]. The rapid rise in O_3_ levels outside of the summer season can enhance atmospheric oxidative capacity, potentially leading to the increased formation of secondary PM_2.5_, including nitrate, sulfate, and organic components.

### 3.2. Identifying Key Factors Using RF Models

**Figure 4:**
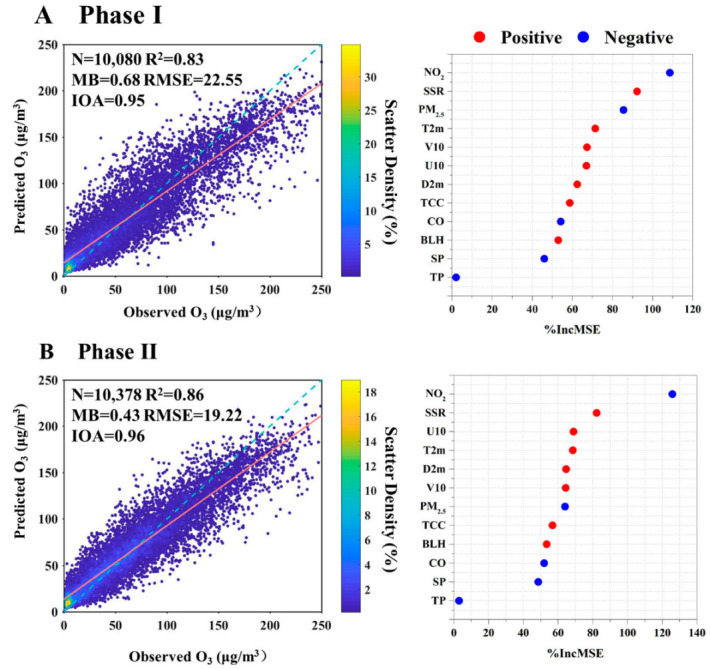
Model performance and variable importance for two phases: (**A**) Phase I, (**B**) Phase II. Cross-validated models R^2^ and MB, RMSE, and IOA are calculated by using a fivefold cross-validation modeling performance for 1 h O_3_ concentration. The orange line and blue dotted line represent the fitted and 1:1 line. The variables are listed in the order of importance from top to bottom. The horizontal axis represents the aggregated increase in the mean squared errors (%IncMSE) from the RF model. A larger value represents higher importance. The correlation relationships (positive and negative) of O_3_ with the variables are identified.

The RF model was employed to predict O_3_ concentrations for both Phase I and Phase II. As shown in Figure S3, during the training phase, the model explained 83% and 86% of the measured O_3_ for Phase I and Phase II, respectively. The RMSE was 9.55 and 7.96 μg m^−3^ for Phase I and Phase II, respectively. The performances of the testing dataset in RF model for the two phases are shown in Figure 4. For both phases, the values of MB were minor, and the values of R^2^ and IOA were close to 1. The slope and intercept values were 0.77 and 14.25 for Phase I and 0.79 and 13.49 for Phase II. It is noteworthy that the RF model tended to underestimate and overestimate O_3_ concentrations at relatively high and low values, resulting in relatively higher RMSE for both phases. This discrepancy can be attributed to the RF model’s tendency to exhibit larger biases in predicting extreme values due to the absence of certain O_3_ precursor data, such as VOC [29,54]. Nevertheless, the RF model could successful reproduce O_3_ concentration using the selected factors.

As shown in Figure 4, the top 5 factors for Phase I were NO_2_, SSR, PM_2.5_, T2m, and V10. The photolysis of NO_2_ produced an oxygen atom, and O_3_ was then produced from the combination of the oxygen atom and O_2_ [1]. Hence, NO_2_ and SSR were the notably influential factors in O_3_ formation. PM_2.5_ was identified as the third most significant factor contributing to O_3_ formation in Phase I. The reduction of PM_2.5_ during this phase may elevate O_3_ concentrations through modulations in atmospheric heterogeneous reaction kinetics, solar radiation-driven photolysis efficiencies, and planetary boundary layer transport dynamics [20,22]. Temperature was also an important factor influencing O_3_ formation in Phase I. Chemical kinetics rates involved in O_3_ production increased with the increase of temperature [55]. Additionally, the VOC emissions, including biogenic emission rates and anthropogenic emissions (such as solvent evaporation), may be enhanced in hot weather [56,57].

In Phase II, NO_2_ and SSR remained prominent factors, indicating the local formation of O_3_. Other high-ranking variables were predominantly meteorology-related, including U10, D2m, T2m, and V10. Unlike in Phase I, PM_2.5_ was less important due to the relatively smaller change in the concentrations in Phase II. O_3_ enhancement due to PM_2.5_ dropping significantly depends on the current level of PM_2.5_ concentration and its decline magnitude [22,23]. The decreased amplitude and the level of PM_2.5_ concentrations were smaller in Phase II, resulting in less importance of PM_2.5_ in O_3_ formation. Although PM_2.5_ ranked seventh in Phase II, its %IncMSE value was close to the high-ranking meteorology-related factors—higher than BLH and CO. In addition, the concentration of PM_2.5_ was at a high level (about 51 μg m^−3^ in 2022), exceeding Class I limit values of the National Ambient Air Quality Standard (NAAQS) (35 μg m^−3^). Hence, PM_2.5_ remains a significant factor in O_3_ formation in recent years for Xinxiang City and also for the other cities with a high PM_2.5_ concentration.

### 3.3. Role of PM_2.5_ in O_3_ Formation

From 19 June to 25 June 2021, the average O_3_ concentration (128.63 μg m^−3^) was in excess of the CNAAQS 1 h mass-based standards of 120 μg m^−3^. The period was identified as being traceable to an O_3_ episode. During the episode, a high level of O_3_ concentration (up to 257 μg m^−3^) was observed. The concentrations of SO_2_, NO, NO_2_, and CO were 13.60, 3.64, 32.46 μg m^−3^, and 0.50 mg m^−3^ on average. The PM_2.5_ concentration was at a relatively low level, with 25.00 μg m^−3^ being the average. The average mixing ratios of 35 NMVOCs are summarized in Table S2. The other information about the O_3_ episode is also introduced in the Supplementary Materials.

The identified O_3_ episode (19 June to 25 June 2021) was used by OBM for a simulation study. The comparison of observed and simulated O_3_ during the identified O_3_ episode is shown in Figure S4. The model accurately captured the diurnal profile of O_3_, demonstrating satisfactory performance. The average concentration of observed and simulated O_3_ during the episode was 128.09 and 128.63 μg m^−3^, respectively, with a high R^2^ value of 0.96. In addition, the MB, RMSE, and IOA were 0.82 μg m^−3^, 8.08 μg m^−3^, and 0.97, respectively, further validating the model’s capability to reproduce the variations of O_3_ effectively and enabling its use for subsequent analysis.

As mentioned in Section 2.3, the heterogeneous reactions and changing of photolysis rates linked to PM_2.5_ were incorporated into the model. Hence, we conducted experiments to assess how variations in PM_2.5_ concentration affected O_3_ levels. During the episode, the concentration of PM_2.5_ was relatively low, with about 25 μg m^−3^ on average. To illustrate concrete situations of pollution, O_3_ concentration was simulated by OBM with a series of PM_2.5_ concentrations (0–3 times PM_2.5_ concentration). The diurnal profile of O_3_ concentration under different PM_2.5_ concentrations is shown in Figure S5. The O_3_ concentration rose with the dropping of the PM_2.5_ concentration. The maximum disparity in O_3_ concentration under different PM_2.5_ concentrations reached up to 32.46 μg m^−3^ (Figure S6), indicating the significant impact of PM_2.5_ on O_3_ formation. In addition, the rangeabilities of O_3_ under difference PM_2.5_ concentrations was higher in the daytime and lower at nighttime. The reduction of HO_2_ by heterogeneous loss in PM_2.5_ was the major mechanism at nighttime. The decrease in PM_2.5_ could lead to an increasing HO_2_ concentration due to less HO_2_ heterogeneous loss on the ambient aerosol [22]. The NO titration effect on O_3_ could be offset by an elevated HO_2_ concentration [11]. During the daytime, enhanced photolysis rates resulting from decreased PM_2.5_ concentration further facilitated O_3_ formation. Both mechanisms played significant roles in O_3_ formation during the daytime.

**Figure 5:**
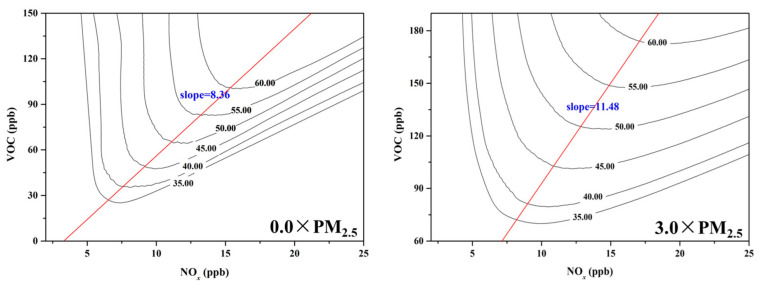
The EKMA under different PM_2.5_ concentrations.

The Empirical Kinetic Modeling Approach (EKMA) diagram can categorize O_3_ formation into either “NO_x_ limited” or “VOC limited” regime [20], providing a basis for effective O_3_ pollution control policies. To investigate the impact of PM_2.5_ on O_3_ pollution control strategies, EKMA curves were constructed by OBM under both 0.0 × PM_2.5_ and 3.0 × PM_2.5_ scenarios (Figure 5). The EKMA curve was changed under different concentrations of PM_2.5_, with the slope of the ridgeline (VOC/NO_x_) increasing from 8.36 under 0.0 × PM_2.5_ scenarios to 11.48 under 3.0 × PM_2.5_ scenarios. The result meant that the O_3_ formation regime tended to “NO_x_ limited” with the dropping of PM_2.5_ concentration. PM_2.5_ has a great impact on the O_3_ sensitivity regime, thereby affecting the production rate of surface O_3_. The aerosol chemistry and photochemistry were the main mechanism for the shift of O_3_ chemical regimes under different PM_2.5_ concentrations [58]. As previously discussed, the concentration of the HO_2_ concentration increases as the level of PM_2.5_ declines, accelerating the RO_x_ cycle (OH→RO→RO_2_→HO_2_→OH) with peroxyl-radical self-reactions predominating under these conditions. Therefore, when formulating policies for VOC and NO_x_ emission reductions to control O_3_ pollution, it is crucial to pay more attention to changes in PM_2.5_ concentration [44].

### 3.4. Limitations

In the present study, a comprehensive dataset from field measurements was employed as a model constraint. The dataset included key parameters, such as the concentrations of reactive species, mixing layer height, and photolysis frequencies. Additionally, the state-of-the-art gas chemistry mechanism (MCM) was used in the OBM. The uncertainties of the OBM were mainly determined by the complexities of atmospheric “Haze Chemistry” [59]. Multiple heterogeneous reactions coexisted on the aerosol surfaces [60,61]. Therefore, some heterogeneous reaction might have an impact on O_3_ production, such as the heterogeneous formation of HONO and HNO_3_ [62] and heterogeneous loss of O_3_ [63]. However, the heterogeneous reactions mechanism was unrevealed, with a big range of heterogeneous uptake coefficients [11,22]. Hence, only the heterogeneous reaction of HO_2_ on aerosols surfaces, the paramount heterogeneous reaction impacting O_3_ formation, was considered in our study.

## 4. Conclusions

Clean air actions have been implemented by the Chinese government to improve the severe air pollution issue since 2013. However, the increasing trend of O_3_ has been inconsistent with the decline of PM_2.5_ in China. In Xinxiang City, the O_3_ concentration increased by the rate of 3.41 μg m^−3^ yr^−1^ from 2015 to 2022. This increase can be divided into two phases: Phase I (2015–2018) saw a high rate of increase (7.89 μg m^−3^), while Phase II (2019–2022) experienced a lower rate (2.89 μg m^−3^). The O_3_ pollution from warm seasons should be paid more attention, due to the steady increasing O_3_ concentration in autumn and winter since 2018. The developed RF model effectively simulated O_3_ concentrations, identifying NO_2_ and surface net solar radiation as primary factors in O_3_ formation for both phases. In Phase I, PM_2.5_ ranked third in O_3_ formation, while in Phase II, PM_2.5_ remained a significant factor due to its persistently high concentration in Xinxiang City. The OBM incorporated into MCM was used to explore how PM_2.5_ influences O_3_ formation. The O_3_ concentration was raised with the dropping of PM_2.5_ by the process of the heterogeneous reaction and photolysis rates. The O_3_ formation regime tended to “NO_x_ limited” with the dropping of the PM_2.5_ concentration. Neglecting the role of PM_2.5_ in O_3_ formation could have adverse effects on O_3_ pollution control policies. Further research into heterogeneous uptake coefficients would be beneficial in reducing the uncertainties associated with heterogeneous reactions in real atmospheric aerosols. Our results provide powerful evidence for on-going coordinated control of O_3_ and PM_2.5_ in a typical city of the Central Plains urban agglomeration.

## Figures and Tables

**Table 1:** The main information of the nine meteorological variables.

Abbreviations	Names of Variable	Unit
U10	10 m u-component of wind	m·s^−1^
V10	10 m v-component of wind	m·s^−1^
D2m	2 m dewpoint temperature	K
T2m	2 m temperature	K
BLH	Boundary layer height	m
SSR	Surface net solar radiation	J·m^−2^
SP	Surface pressure	Pa
TCC	Total cloud cover	Dimensionless
TP	Total precipitation	cm

## Data Availability

The data that support the findings of this study are available from the corresponding author upon reasonable request.

## Supplementary Materials

The following supporting information can be downloaded at: https://www.mdpi.com/article/10.3390/toxics13050330/s1, Text S1: Details setting in OBM; Figure S1: The error rate curve of RF model by fivefold cross-validation method; Figure S2: The simulated O_3_ concentrations with different uptake coefficient of HO_2_ (γHO_2_) on aerosols; Figure S3: The performances of the training dataset in RF model for Phase I and Phase II; Figure S4: The comparison of simulated and observed O_3_ concentrations; Figure S5: The diurnal profile of O_3_ under a series of PM_2.5_ concentrations; Figure S6: The simulated maximum O_3_ concentration by OBM under different PM_2.5_ concentrations; Table S1: Parameters tuned for RF model; Table S2: The average concentration of VOC during the sampling period.

## References

[1] Lyu X., Li K., Guo H., Morawska L., Zhou B., Zeren Y., Jiang F., Chen C., Goldstein A.H., Xu X. (2023). A synergistic ozone-climate control to address emerging ozone pollution challenges. One Earth.

[2] Liu Y., Geng G., Cheng J., Liu Y., Xiao Q., Liu L., Shi Q., Tong D., He K., Zhang Q. (2023). Drivers of Increasing Ozone during the Two Phases of Clean Air Actions in China 2013–2020. Environ. Sci. Technol..

[3] Wang W., Parrish D.D., Wang S., Bao F., Ni R., Li X., Yang S., Wang H., Cheng Y., Su H. (2022). Long-term trend of ozone pollution in China during 2014–2020: Distinct seasonal and spatial characteristics and ozone sensitivity. Atmos. Chem. Phys..

[4] Zhang L., Wang L.L., Liu B.Y., Tang G.Q., Liu B.X., Li X., Sun Y., Li M.G., Chen X.Y., Wang Y.S. (2023). Contrasting effects of clean air actions on surface ozone concentrations in different regions over Beijing from May to September 2013–2020. Sci. Total Environ..

[5] Qian J., Liao H., Yang Y., Li K., Chen L., Zhu J. (2022). Meteorological influences on daily variation and trend of summertime surface ozone over years of 2015-2020: Quantification for cities in the Yangtze River Delta. Sci. Total Environ..

[6] Wu K., Wang Y.R., Qiao Y.H., Liu Y.M., Wang S.G., Yang X.Y., Wang H.L., Lu Y.Q., Zhang X.L., Lei Y. (2022). Drivers of 2013–2020 ozone trends in the Sichuan Basin, China: Impacts of meteorology and precursor emission changes. Environ. Pollut..

[7] Lu X., Zhang L., Wang X.L., Gao M., Li K., Zhang Y.Z., Yue X., Zhang Y.H. (2020). Rapid Increases in Warm-Season Surface Ozone and Resulting Health Impact in China Since 2013. Environ. Sci. Technol. Lett..

[8] Chen X., Jiang Z., Shen Y., Li R., Fu Y., Liu J., Han H., Liao H., Cheng X., Jones D.B.A. (2021). Chinese Regulations Are Working—Why Is Surface Ozone Over Industrialized Areas Still High? Applying Lessons From Northeast US Air Quality Evolution. Geophys. Res. Lett..

[9] Li K., Jacob D.J., Shen L., Lu X., Smedt I.D., Liao H., PHYSICS (2020). Increases in surface ozone pollution in China from 2013 to 2019: Anthropogenic and meteorological influences. Atmos. Chem. Phys..

[10] Wang T., Xue L.K., Brimblecombe P., Lam Y.F., Li L., Zhang L. (2017). Ozone pollution in China: A review of concentrations, meteorological influences, chemical precursors, and effects. Sci. Total Environ..

[11] Tan Z., Hofzumahaus A., Lu K., Brown S.S., Holland F., Huey L.G., Kiendler-Scharr A., Li X., Liu X., Ma N. (2020). No Evidence for a Significant Impact of Heterogeneous Chemistry on Radical Concentrations in the North China Plain in Summer 2014. Environ. Sci. Technol..

[12] Li M., Wang T., Shu L., Qu Y., Xie M., Liu J., Wu H., Kalsoom U. (2021). Rising surface ozone in China from 2013 to 2017: A response to the recent atmospheric warming or pollutant controls?. Atmos. Environ..

[13] Ding J., Dai Q., Fan W., Lu M., Zhang Y., Han S., Feng Y. (2023). Impacts of meteorology and precursor emission change on O_3_ variation in Tianjin, China from 2015 to 2021. J. Environ. Sci..

[14] Xiong K., Xie X., Mao J., Wang K., Huang L., Li J., Hu J. (2023). Improving the accuracy of O_3_ prediction from a chemical transport model with a random forest model in the Yangtze River Delta region, China. Environ. Pollut..

[15] Li L., Xie F., Li J., Gong K., Xie X., Qin Y., Qin M., Hu J. (2022). Diagnostic analysis of regional ozone pollution in Yangtze River Delta, China: A case study in summer 2020. Sci. Total Environ..

[16] Shi Z., Huang L., Li J., Ying Q., Hu J. (2020). Sensitivity analysis of the surface ozone and fine particulate matter to meteorological parameters in China. Atmos. Chem. Phys..

[17] Qin Y., Li J., Gong K., Wu Z., Chen M., Qin M., Huang L., Hu J. (2021). Double high pollution events in the Yangtze River Delta from 2015 to 2019: Characteristics, trends, and meteorological situations. Sci. Total Environ..

[18] Weng X., Forster G.L., Nowack P. (2022). A machine learning approach to quantify meteorological drivers of ozone pollution in China from 2015 to 2019. Atmos. Chem. Phys..

[19] Tan Z., Lu K., Ma X., Chen S., He L., Huang X., Li X., Lin X., Tang M., Yu D. (2022). Multiple Impacts of Aerosols on O_3_ Production Are Largely Compensated: A Case Study Shenzhen, China. Environ. Sci. Technol..

[20] Ivatt P.D., Evans M.J., Lewis A.C. (2022). Suppression of surface ozone by an aerosol-inhibited photochemical ozone regime. Nat. Geosci..

[21] Dyson J.E., Whalley L.K., Slater E.J., Woodward-Massey R., Ye C.X., Lee J.D., Squires F., Hopkins J.R., Dunmore R.E., Shaw M. (2023). Impact of HO_2_ aerosol uptake on radical levels and O_3_ production during summertime in Beijing. Atmos. Chem. Phys..

[22] Li K., Jacob D.J., Liao H., Shen L., Zhang Q., Bates K.H. (2019). Anthropogenic drivers of 2013–2017 trends in summer surface ozone in China. Proc. Natl. Acad. Sci. USA.

[23] Shao M., Wang W., Yuan B., Parrish D.D., Li X., Lu K., Wu L., Wang X., Mo Z., Yang S. (2021). Quantifying the role of PM_2.5_ dropping in variations of ground-level ozone: Inter-comparison between Beijing and Los Angeles. Sci. Total Environ..

[24] Hou L., Dai Q., Song C., Liu B., Guo F., Dai T., Li L., Liu B., Bi X., Zhang Y. (2022). Revealing Drivers of Haze Pollution by Explainable Machine Learning. Environ. Sci. Technol. Lett..

[25] Vu T.V., Shi Z., Cheng J., Zhang Q., He K., Wang S., Harrison R.M. (2019). Assessing the impact of clean air action on air quality trends in Beijing using a machine learning technique. Atmos. Chem. Phys..

[26] Wang L., Zhao Y., Shi J., Ma J., Liu X., Han D., Gao H., Huang T. (2023). Predicting ozone formation in petrochemical industrialized Lanzhou city by interpretable ensemble machine learning. Environ. Pollut..

[27] Watson G.L., Telesca D., Reid C.E., Pfister G.G., Jerrett M. (2019). Machine learning models accurately predict ozone exposure during wildfire events. Environ. Pollut..

[28] Ma R., Ban J., Wang Q., Zhang Y., Yang Y., He M.Z., Li S., Shi W., Li T. (2021). Random forest model based fine scale spatiotemporal O_3_ trends in the Beijing-Tianjin-Hebei region in China, 2010 to 2017. Environ. Pollut..

[29] Yang J., Wen Y., Wang Y., Zhang S., Pinto J.P., Pennington E.A., Wang Z., Wu Y., Sander S.P., Jiang J.H. (2021). From COVID-19 to future electrification: Assessing traffic impacts on air quality by a machine-learning model. Proc. Natl. Acad. Sci. USA.

[30] Zhan J., Liu Y., Ma W., Zhang X., Wang X., Bi F., Zhang Y., Wu Z., Li H. (2022). Ozone formation sensitivity study using machine learning coupled with the reactivity of volatile organic compound species. Atmos. Meas. Tech..

[31] Zhu J., Wang S., Wang H., Jing S., Lou S., Saiz-Lopez A., Zhou B. (2020). Observationally constrained modeling of atmospheric oxidation capacity and photochemical reactivity in Shanghai, China. Atmos. Chem. Phys..

[32] Edwards P.M., Brown S.S., Roberts J.M., Ahmadov R., Banta R.M., deGouw J.A., Dubé W.P., Field R.A., Flynn J.H., Gilman J.B. (2014). High winter ozone pollution from carbonyl photolysis in an oil and gas basin. Nature.

[33] Jia C., Tong S., Zhang X., Li F., Zhang W., Li W., Wang Z., Zhang G., Tang G., Liu Z. (2023). Atmospheric oxidizing capacity in autumn Beijing: Analysis of the O_3_ and PM_2.5_ episodes based on observation-based model. J. Environ. Sci..

[34] Michoud V., Kukui A., Camredon M., Colomb A., Borbon A., Miet K., Aumont B., Beekmann M., Durand-Jolibois R., Perrier S. (2012). Radical budget analysis in a suburban European site during the MEGAPOLI summer field campaign. Atmos. Chem. Phys..

[35] Li Z., Xue L., Yang X., Zha Q., Tham Y.J., Yan C., Louie P.K.K., Luk C.W.Y., Wang T., Wang W. (2018). Oxidizing capacity of the rural atmosphere in Hong Kong, Southern China. Sci. Total Environ..

[36] Ling Z.H., Guo H., Lam S.H.M., Saunders S.M., Wang T. (2015). Atmospheric photochemical reactivity and ozone production at two sites in Hong Kong: Application of a Master Chemical Mechanism–photochemical box model. J. Geophys. Res.-Atmos..

[37] Balamurugan V., Balamurugan V., Chen J. (2022). Importance of ozone precursors information in modelling urban surface ozone variability using machine learning algorithm. Sci. Rep..

[38] Gong X., Hong S., Jaffe D.A. (2018). Ozone in China: Spatial Distribution and Leading Meteorological Factors Controlling O_3_ in 16 Chinese Cities. Aerosol Air Qual. Res..

[39] Hu C., Kang P., Jaffe D.A., Li C., Zhou M. (2021). Understanding the impact of meteorology on ozone in 334 cities of China. Atmos. Environ..

[40] Brancher M. (2021). Increased ozone pollution alongside reduced nitrogen dioxide concentrations during Vienna’s first COVID-19 lockdown: Significance for air quality management. Environ. Pollut..

[41] Jenkin M.E., Young J.C., Rickard A.R. (2015). The MCM v3.3.1 degradation scheme for isoprene. Atmos. Chem. Phys..

[42] Lin C., Li Y., Yuan Z., Lau A.K.H., Li C., Fung J.C.H. (2015). Using satellite remote sensing data to estimate the high-resolution distribution of ground-level PM_2.5_. Remote Sens. Environ..

[43] Zhao S., Hu B., Du C., Liu H., Wang Y. (2021). Photolysis rate in the Beijing-Tianjin-Hebei region: Reconstruction and long-term trend. Atmos. Res..

[44] Zhao S., Hu B., Liu H., Du C., Wang Y. (2021). The influence of aerosols on the NO_2_ photolysis rate in a suburban site in North China. Sci. Total Environ..

[45] Jenkin M.E., Saunders S.M., Wagner V., Pilling M.J. (2003). Protocol for the development of the Master Chemical Mechanism, MCM v3 (Part A): Tropospheric degradation of non-aromatic volatile organic compounds. Atmos. Chem. Phys..

[46] Xue L.K., Wang T., Gao J., Ding A.J., Zhou X.H., Blake D.R., Wang X.F., Saunders S.M., Fan S.J., Zuo H.C. (2014). Ground-level ozone in four Chinese cities: Precursors, regional transport and heterogeneous processes. Atmos. Chem. Phys..

[47] Wang W., Parrish D., Li X., Shao M., Liu Y., Mo Z., Lu S., Hu M., Fang X., Wu Y. (2020). Exploring the drivers of the increased ozone production in Beijing in summertime during 2005–2016. Atmos. Chem. Phys..

[48] Gao W., Tie X.X., Xu J.M., Huang R.J., Mao X.Q., Zhou G.Q., Chang L.Y. (2017). Long-term trend of O_3_ in a mega City (Shanghai), China: Characteristics, causes, and interactions with precursors. Sci. Total Environ..

[49] Li X.B., Yuan B., Parrish D.D., Chen D.H., Song Y.X., Yang S.X., Liu Z.J., Shao M. (2022). Long-term trend of ozone in southern China reveals future mitigation strategy for air pollution. Atmos. Environ..

[50] Li K., Jacob D., Liao H., Zhu J., Shah V., Shen L., Bates K., Zhang Q., Zhai S. (2019). A two-pollutant strategy for improving ozone and particulate air quality in China. Nat. Geosci..

[51] Ma Z., Xu J., Quan W., Zhang Z., Lin W., Xu X. (2016). Significant increase of surface ozone at a rural site, north of eastern China. Atmos. Chem. Phys..

[52] Jia C., Wang Y., Li Y., Huang T., Mao X., Mo J., Li J., Wanyanhan J., Liang X., Gao H. (2018). Oxidative Capacity and Radical Chemistry in a Semi-arid and Petrochemical-industrialized City, Northwest China. Aerosol Air Qual. Res..

[53] Li K., Jacob D.J., Liao H., Qiu Y., Shen L., Zhai S., Bates K.H., Sulprizio M.P., Song S., Lu X. (2021). Ozone pollution in the North China Plain spreading into the late-winter haze season. Proc. Natl. Acad. Sci. USA.

[54] Robinson M.C., Glen R.C., Lee A.A. (2020). Validating the validation: Reanalyzing a large-scale comparison of deep learning and machine learning models for bioactivity prediction. J. Comput. Aid. Mol. Des..

[55] Bloomer B.J., Stehr J.W., Piety C.A., Salawitch R.J., Dickerson R.R. (2009). Observed relationships of ozone air pollution with temperature and emissions. Geophys. Res. Lett..

[56] Gao M., Wang F., Ding Y.H., Wu Z.W., Xu Y.Y., Lu X., Wang Z.F., Carmichael G.R., McElroy M.B. (2023). Large-scale climate patterns offer preseasonal hints on the co- occurrence of heat wave and O_3_ pollution in China. Proc. Natl. Acad. Sci. USA.

[57] Wang H.L., Wu K., Liu Y.M., Sheng B.S., Lu X., He Y.P., Xie J.L., Wang H.C., Fan S.J. (2021). Role of Heat Wave-Induced Biogenic VOC Enhancements in Persistent Ozone Episodes Formation in Pearl River Delta. J. Geophys. Res.-Atmos..

[58] Liu C., Liang J., Li Y., Shi K. (2023). Fractal analysis of impact of PM_2.5_ on surface O_3_ sensitivity regime based on field observations. Sci. Total Environ..

[59] Biwu C., Qingxin M., Fengkui D., Jinzhu M., Jingkun J., Kebin H., Hong H. (2020). Atmospheric “Haze Chemistry”: Concept and Research Prospects. Prog. Chem..

[60] Jacob D.J. (2000). Heterogeneous chemistry and tropospheric ozone. Atmos. Environ..

[61] Liu Y., He G., Chu B., Ma Q., He H. (2023). Atmospheric heterogeneous reactions on soot: A review. Fundam. Res..

[62] Li Q.Y., Zhang L., Wang T., Wang Z., Fu X., Zhang Q. (2018). “New” Reactive Nitrogen Chemistry Reshapes the Relationship of Ozone to Its Precursors. Environ. Sci. Technol..

[63] Kamm S., Möhler O., Naumann K.H., Saathoff H., Schurath U. (1999). The heterogeneous reaction of ozone with soot aerosol. Atmos. Environ..

