# Supplementary material for: Evidence for Coordinated Control of PM2.5 and O3: Long-Term Observational Study in a Typical City of Central Plains Urban Agglomeration
Source: Toxics. 2025 Apr 23;13(5):330. doi: 10.3390/toxics13050330 (PMC12116060; doi:10.3390/toxics13050330)
Supplement: Supplementary file 1 [file toxics-13-00330-s001.zip › toxics-3552337-supplementary.pdf]

Supporting Information (SI):

Evidence for Coordinated Control of $PM_{2.5}$ and O_3: Long-Term Observational Study in a Typical City of Central Plains Urban Agglomeration

Chenhui Jia [1], Guangxuan Yan [1], Xinyi Yu [1], Xue Li [1], Jing Xue [2], and Yanan Wang [3,*] and Zhiguo Cao [1,*]

1 School of Environment, Key Laboratory for Yellow River and Huai River Water Environment and Pollution Control, Ministry of Education, Henan Normal University, Xinxiang 453007, China

2 Key Laboratory for Space Bioscience and Biotechnology, School of Life Sciences, Northwestern Polytechnical University, Xi'an 710072, China

3 Department of Civil and Environmental Engineering, The Hong Kong Polytechnic University, Hong Kong, China

* Correspondence: yanan.wang@connect.polyu.hk (Y.W.); wq11ab@163.com (Z.C.)

Details setting in OBM

Photolysis frequencies of NO_2 (JNO_2) are affected by aerosols, temperature, solar zenith angle (SZA), clouds, and so on. JNO_2 and aerosol optical depth (AOD) presented logarithmic relations under different solar zenith angle (SZA) levels, and the aerosol attenuation effect on JNO_2 decreased as AOD increased [1,2]. In our study, The Tropospheric Ultraviolet and Visible (TUV) radiation model (version 5.3) was used to calculate JNO_2 in our study. TUV used the discrete-ordinate algorithm (DISORT) with four streams and calculated the actinic flux spectra with a wavelength range of 280-420 nm in 1 nm steps and resolution. The calculated aerosol optical depth (AOD), according to measured $PM_{2.5}$, RH, and atmosphere boundary layer height, was constrained in the TUV model. The calculated AOD (0.32) from June 19 to June 25, 2021 was comparable with the satellite-derived AOD (0.35) (MERRA-2, aerosol optical thickness (AOT) at 550 nm, https://disc.gsfc.nasa.gov/datasets/M2T1NXAER_5.12.4/summary). The single scattering albedo (SSA) and Ångström exponent (AE) was set to 0.9 and 1.07 during the episode. Thus, the parametric relationship between JNO_2 and $PM_{2.5}$ was established, and further input into the OBM as one of the constraints.

There was a parametric relationship between the aerosol surface concentration (S_{aero}) and $PM_{2.5}$. The fitted linear relationship between S_{aero} and $PM_{2.5}$ in Beijing was established based on the measurement during 2006–2016 [3], as shown in Equation (1):

$$S_{aero} = 8.9 \times PM_{2.5} + 259 \qquad (1)$$

where, the units of $PM_{2.5}$ and Sa were μg m^{-3} and μm^{2} cm^{-2}. The equation was applied in Xinxiang City, due to the similar atmospheric environment in Beijing and Xinxiang.

Hence, S_{aero} was calculated by the measured $PM_{2.5}$ in Xinxiang City. The calculated S_{aero} was also input into the OBM as one of the constraints.

Details of the episode

During the episode, the wind speed of the episode was generally below 2 m Sec^{-1}. The stable weather condition implied that the regional transportation played minor roles in the air pollutants. The selected episode could represent and reflect the air pollution caused by local chemistry in Xinxiang City. Hence, the OBM was the most appropriate tool for investigating the chemical processes of the selected O_3 episode.

Table S1. Parameters tuned for RF model.

Parameter	Value
Number of trees	500
Maximum tree depth	20
The minimum number of samples required to split an internal node	2
The minimum number of samples required to be at a leaf node	1

Table S2. The average concentration of VOC during the sampling period.

Species	Compounds	Concentration (ppb)	Standard Deviation
Alkanes	Ethane	1.48	0.79
	Propane	1.27	0.57
	i-Butane	0.57	0.31
	n-Butane	0.71	0.37
	i-Pentane	2.50	1.73
	n-Pentane	0.43	0.22
	n-Hexane	0.10	0.05
	n-Heptane	0.03	0.02
	n-Octane	0.01	0.01
	n-Nonane	0.01	0.01
	n-Decane	0.01	0.01
	n-Undecane	0.01	0.00
Alkenes	Ethylene	0.39	0.20
	Propylene	0.38	0.14
	1-Butene	0.22	0.11
	Isoprene	0.58	0.56
	1,3-Butadiene	0.04	0.07
Alkynes	Acetylene	0.61	0.40
Aromatics	Benzene	0.39	0.13
	Toluene	0.40	0.26
	Ethylbenzene	0.13	0.08
	m/p-Xylene	0.17	0.10
	Styrene	0.04	0.05
	o-Xylene	0.10	0.06
	m-Ethyltoluene	0.02	0.02
	p-Ethyltoluene	0.01	0.01
	1,3,5-Trimethylbenzene	0.01	0.00
	1,2,4-Trimethylbenzene	0.03	0.02
	1,2,3-Trimethylbenzene	0.01	0.01
Oxygenated compounds	Acetaldehyde	5.20	2.44
	Acrolein	0.09	0.03
	Propanal	0.48	0.50
	Acetone	5.18	3.37
	Hexanal	0.11	0.05
	2-Butanone	0.26	0.06

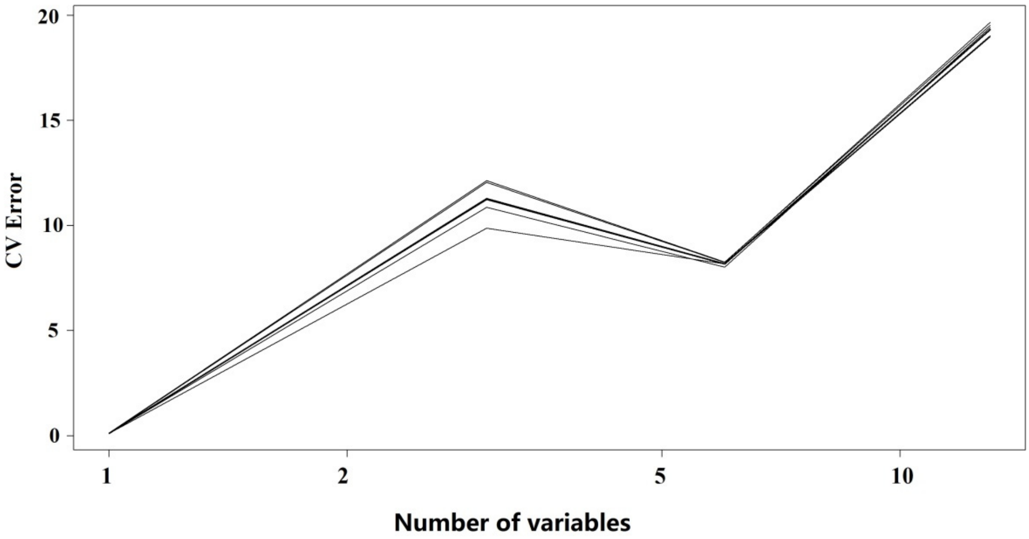

Figure S1. The error rate curve of RF model by fivefold cross-validation method.

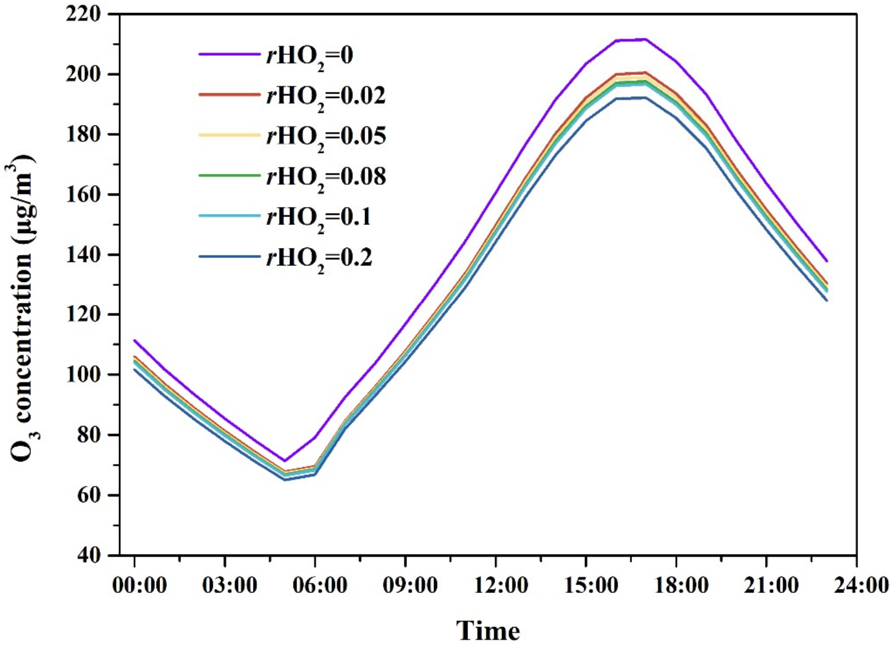

Figure S2. The simulated O_3 concentrations with different uptake coefficientsee of HO_2.

（γHO_2） on aerosols

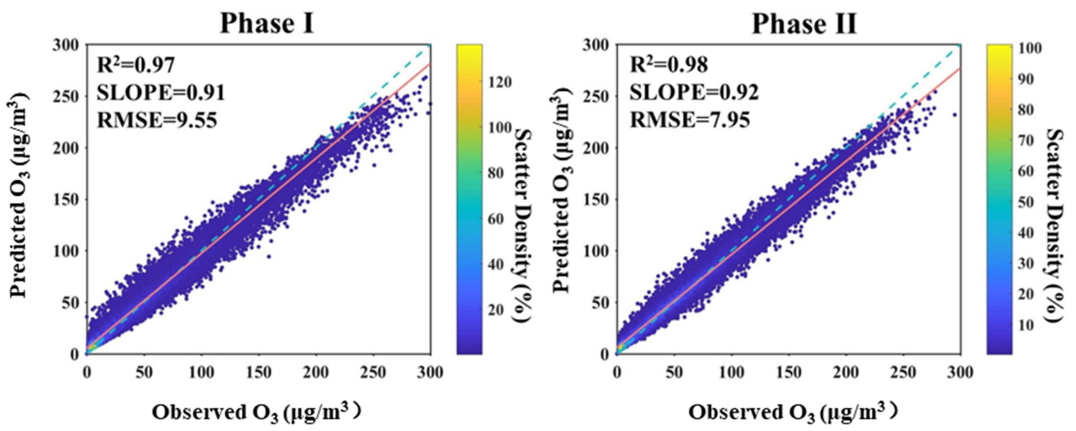

Figure S3. The performances of the training dataset in RF model for Phase I and Phase II.

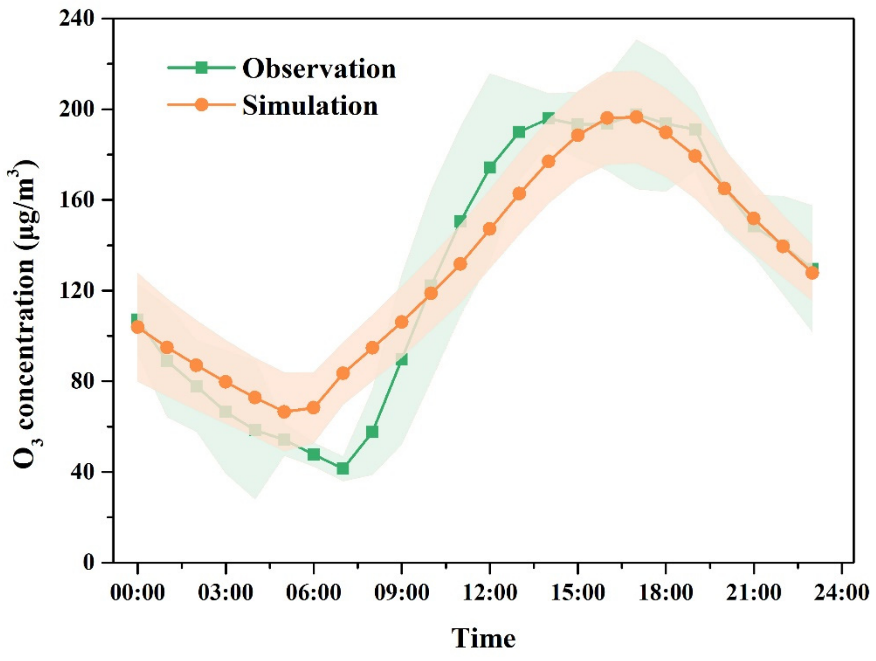

Figure S4. The comparison of simulated and observed O_3 concentrations.

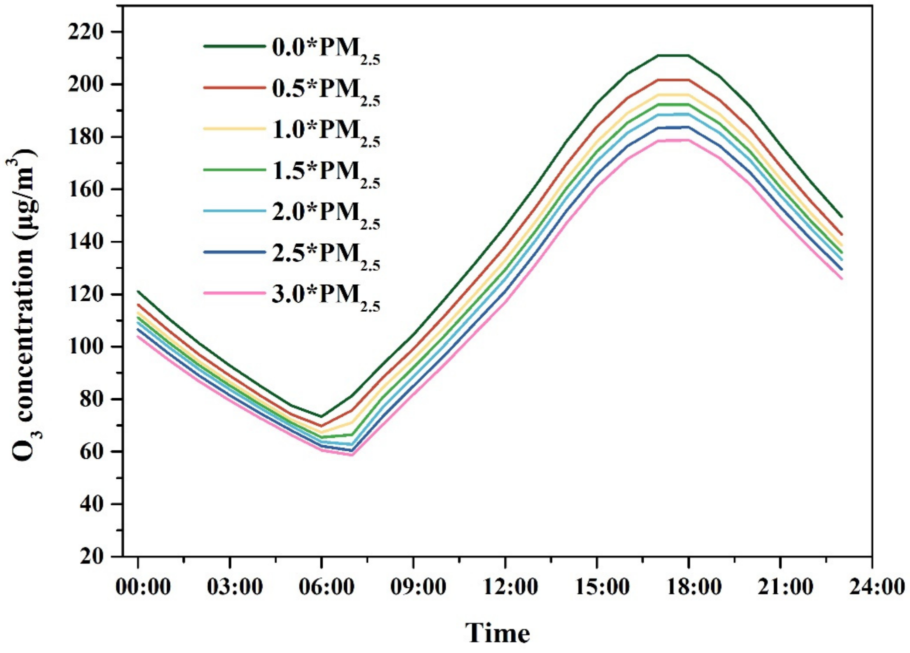

Figure S5. The diurnal profile of O_3 under a series of $PM_{2.5}$ concentrations.

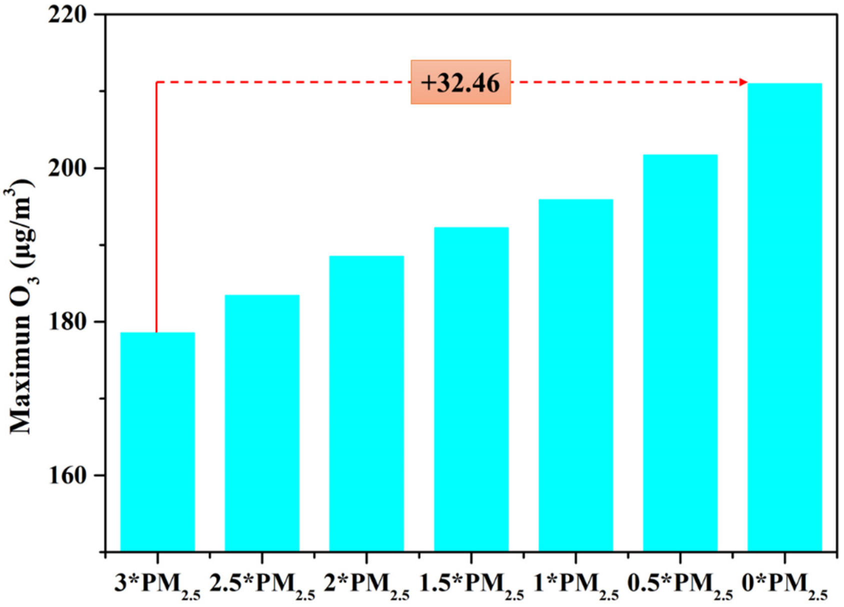

Figure S6. The simulated maximum O_3 concentration by OBM under different $PM_{2.5}$ concentrations.

References:

1. Zhao, S.; Hu, B.; Du, C.; Liu, H.; Wang, Y. Photolysis rate in the Beijing-Tianjin-Hebei region: Reconstruction and long-term trend. *Atmos. Res.* **2021**, *256*, 105568.
2. Zhao, S.; Hu, B.; Liu, H.; Du, C.; Wang, Y. The influence of aerosols on the NO2 photolysis rate in a suburban site in North China. Sci. Total Environ. **2021**, 767, 144788.
3. Shao, M.; Wang, W.; Yuan, B.; Parrish, D.D.; Li, X.; Lu, K.; Wu, L.; Wang, X.; Mo, Z.; Yang, S.; et al. Quantifying the role of PM(2.5) dropping in variations of ground-level ozone: Inter-comparison between Beijing and Los Angeles. Sci. Total Environ. **2021**, 788, 147712. https://doi.org/10.1016/j.scitotenv.2021.147712.
